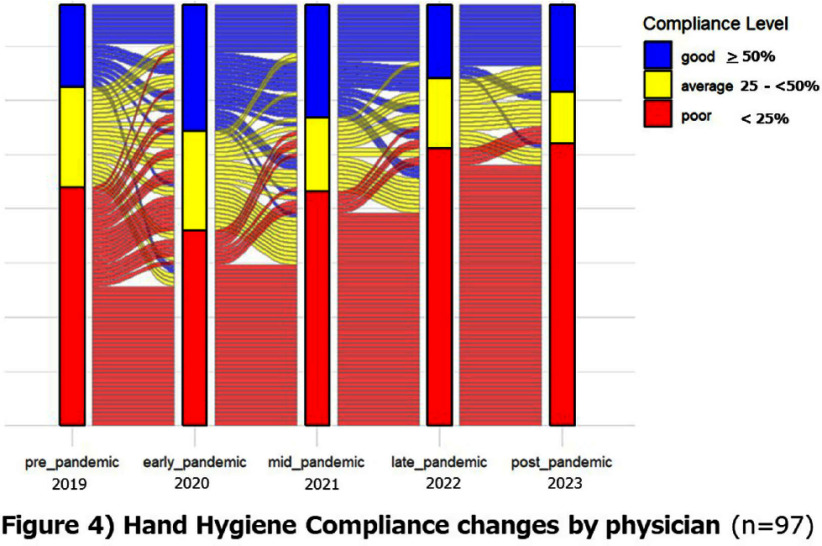# The impact of COVID-19 on the physicians’ hand hygiene adherence during outpatient consultations

**DOI:** 10.1017/ash.2025.338

**Published:** 2025-09-24

**Authors:** Masaki Tanabe, Mikako Tsukawaki, Akie Arai

**Affiliations:** 1 Mie University Hospital

## Abstract

**Background:** Appropriate hand hygiene is one of the most important ways to reduce the transmission of pathogens and prevent healthcare-associated infections (HCAIs), but the rate of compliance among doctors remains low. The purpose of this study is to evaluate the impact of the COVID-19 pandemic on the rate of hand hygiene compliance among doctors during outpatient consultations. **Method:** This study was conducted on doctors providing outpatient care at Mie University Hospital (Japan) from January 2019 to December 2023. The electronic counting device, Hand Hygiene Monitoring System Compleo-IO, was used, which automatically tallies the amount of alcohol-based hand sanitizer used by installing a wireless device under the hand sanitizer dispenser. The hand hygiene compliance rate was calculated by dividing the number of times hand disinfection was performed by the number of patients receiving outpatient care. We measured the hand hygiene compliance rate of each department and each doctor every month, and evaluated the changes in the impact of the pandemic on the hand hygiene compliance rate. In addition, we categorized the compliance rate into poor (0% to < 25%), average (25% to < 50%), and good (50% or more) categories, and visually evaluated the transition of the categories over the years. **Result:** The hand hygiene compliance rate in 27 departments was 24.8% on average before the pandemic (2019), but rose to 35.2-39.4% in the early stages of the pandemic (2021-2022). However, in the late stages of the pandemic (2022-2023), it had returned to baseline values of 25.4-27.2%(figure1, 2). The hand hygiene compliance rate among individual doctors (based on 97 doctors for whom data could be measured continuously over a five-year period) was similarly 26.9% on average before the pandemic, but rose to 32.1% - 37.7% in the early stages of the pandemic. However, in the late stages of the pandemic, it had returned to baseline values of 26.0-26.6%(figure 3,4). **Conclusion:** During the unprecedented COVID-19 pandemic, hand hygiene compliance rates increased in the early stages of the pandemic, but eventually returned to pre-pandemic levels. We hope to use this experience to help us improve compliance rates on an ongoing basis.

**Figure:**
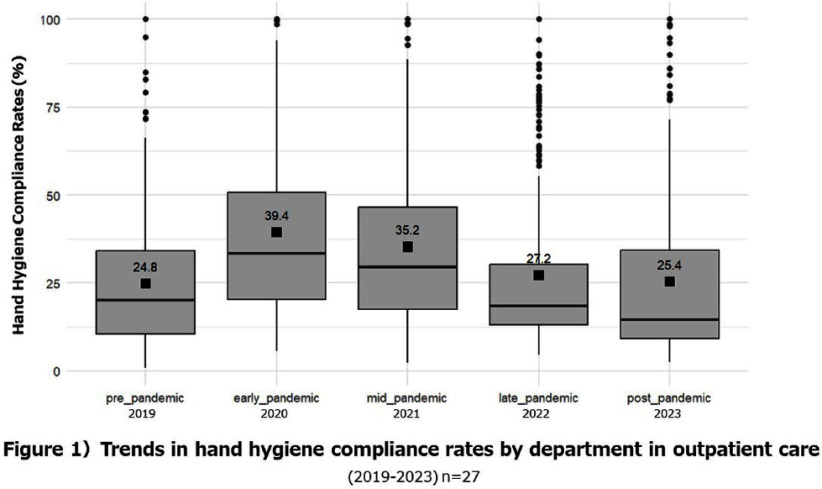

**Figure:**
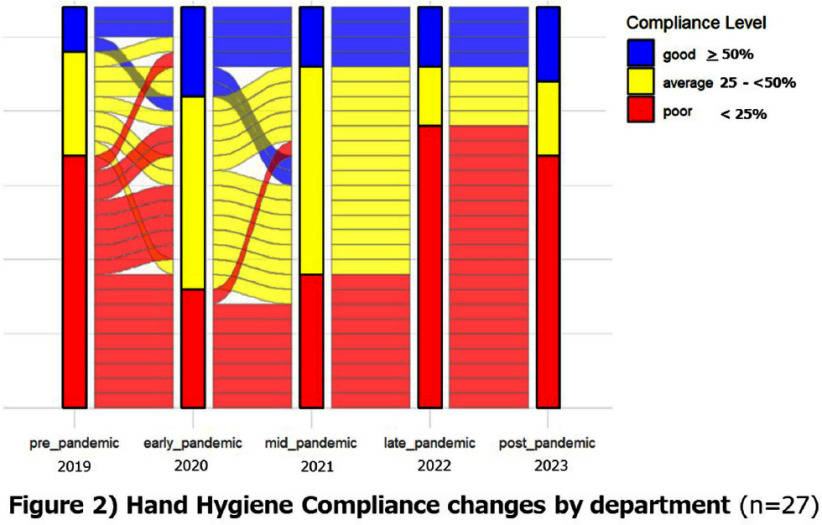

**Figure:**
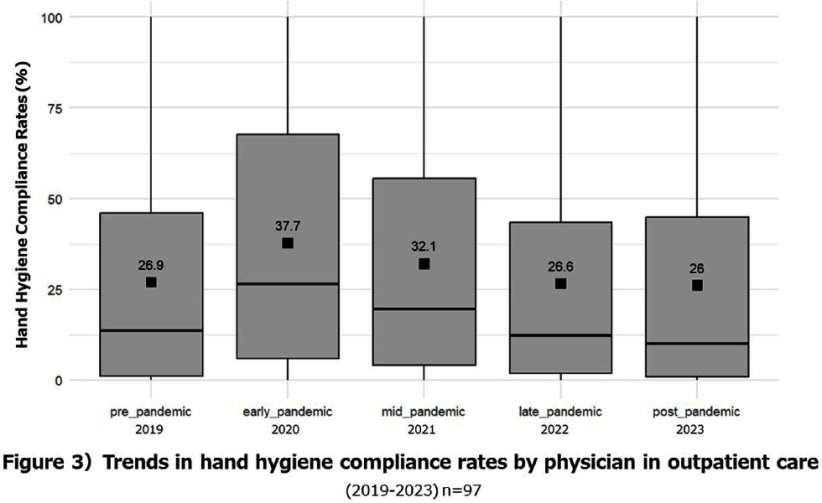

**Figure:**